# Effect of Stretching Force on the Cells of Epithelial Rests of Malassez In Vitro

**DOI:** 10.1155/2010/458408

**Published:** 2010-04-12

**Authors:** Teruyoshi Koshihara, Kenichi Matsuzaka, Toru Sato, Takashi Inoue

**Affiliations:** ^1^Department of Crown & Bridge Prosthodontics, Tokyo Dental College, 1-2-2, Masago, Mihama-ku, Chiba 261-8502, Japan; ^2^Oral Health Science Center hrc7, Department of Clinical Pathophysiology, Tokyo Dental College, 1-2-2, Masago, Mihama-ku, Chiba 261-8502, Japan

## Abstract

*Background and Objective*. The aim of this study was to investigate the behavior of cells from epithelial rest of Malassez (ERM) against stretching force. *Material and Methods*. ERM-cultured cells were stretched for 1 hour, at the cycle of 18% elongation for 1 second followed by 1-second relaxation. The cells without addition of stretching force were used as controls. The cells were observed by immunohistochmical staining using actin 0, 12, 24, 36, 48, and 72 hours. Furthermore, expressions of HSP70-, VEGF-, and OPN-mRNAs of cells were also evaluated using quantitative RT-PCR. *Results*. Actin filaments were randomly orientated in the cytoplasm in the control group, whereas in the stretching group, actin filaments were orientated comparatively parallel to the stretching direction. Expression of HSP70-mRNA in the stretching group was significantly higher than that of control group at 12, 24, 36 hours (*P* < .05). Expression of VEGF-mRNA in the stretching group was significantly higher than that of control group at 24, 36, 48, and 72 hours (*P* < .05). Expression of OPN-mRNA in the stretching group was significantly higher than that of control group at 12 and 24 hours (*P* < .05). *Conclusion*. ERM cells response against the stretching force by expressing HSP70, VEGF, and OPN.

## 1. Introduction

Epithelial-mesenchymal interactions play important roles during tooth formation in the jaw. After the crown formation, the inner and outer enamel epithelium joins together and begins to migrate towards the apical area to form the tooth root. This epithelium is called Hertwig's epithelial root sheath (HERS). HERS cells induce dental papilla cells to differentiate into odontoblasts to form the root dentin and they begin to fragment just before cementogenesis.

After fragmentation of the HERS, some epithelial cells move away from the root surface and remain throughout life in the periodontal ligament. Those cell islands are called epithelial rests of Malassez (ERM) [1–3]. It has been reported that the functions of the ERM are to regulate the width of the periodontal ligament space [4, 5], the growth induction of nerve endings [6], the differentiation of ameloblasts [7], the secretion of enamel protein [8], and the differentiation induction of cementoblasts [5, 9]. Furthermore, the ERM is known to respond to inflammatory cytokines [10], and this may induce increased numbers of cells of the ERM and create either a pocket epithelium [11] or the lining epithelium of the radicular cyst. Moreover, sometimes this process is related to the formation of odontogenic tumors, such as ameloblastomas or odontogenic carcinomas [7, 11, 12].

The periodontal ligament is always exposed to mechanical stress such as compression forces or extension forces during occlusion and chewing. Those mechanical stresses are much milder compared to bridges or denture supported teeth. However, it is known that the periodontal space is kept fixed by instructions from stress immunity factors such as heat shock proteins (HSPs) except in the case of excessive power [13, 14].

It is known that small amounts of HSP exist constantly in all cells, and levels of HSP increase following chemical or mechanical stimulations such as heat [15], oxidative stress [16, 17], acid [18], ischemia, or reperfusion in situ [19]. When a tissue is subjected to stress as described above, HSP initiates biophylactic reactions and acts intracellularly as molecular chaperones and to maintain tissue homeostasis. 

Vascular endothelial growth factor (VEGF) is an essential mediator for angiogenesis [20]. VEGF is produced by many kinds of cells during the vascularization process of developing embryos [21] and during the pathophysiologic processes of ischemia or wound healing [22, 23].

Osteopontin (OPN) is a bone-related protein involved in the production of tooth enamel, dentin, and the cementum. However, it is known that OPN has various other functions elsewhere. For these reasons, HSP, VEGF, and OPN are used as markers for cell responses against biological stress [24].

Morphological molecular studies [25–27] have characterized processes when stretching forces are loaded on periodontal ligament fibroblasts in vivo and in vitro. However, only a few morphological studies have examined the effect of stretching forces on cells of the ERM [28], and no molecular studies have characterized that. 

The purpose of this study was to investigate the behavior of cells derived from the ERM against stretching forces in vitro, and to investigate them at the molecular level.

## 2. Materials and Methods

### 2.1. Cell Culture

Porcine ERM cells were provided by Prof. Abiko, Department of Dental Science, Institute of Personalized Medical Science, Health Sciences University of Hokkaido. ERM cells were cultured in 75 cm^2^ tissue culture flasks (Corning, Tokyo, Japan) with *α*-MEM (Gibco, Carlsbad, CA, USA) containing 10% fetal bovine serum (Sigma, St. Louis, MO, USA) and gentamycin (Sigma). They were cultured by incubation in a humidified atmosphere of 95% air and 5% CO_2_ at 37°C. After the cells became confluent, they were detached using trypsin/ethylenediaminetetraacetic acid (EDTA) (0.25% w/v trypsin/0.02% EDTA, pH 7.2). Subsequently, the cells were resuspended in the supplemented culture medium as described above and used in the following experiments. To identify epithelial cells, Immunofluorescence staining was carried out using a primary antibody against cytokeratin 19 (1 : 100 dilution; Abcam, Cambridge, UK) and a secondary antibody labeled with FITC (1 : 100 dilution; Invitrogen, Carlsbad, CA, USA) and were observed using a laser scanning microscope (LSM5 DUO, Carl Zeiss, Oberkochen, Germany).

### 2.2. Mechanical Stretching

**Figure 1:**
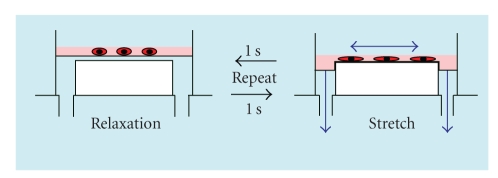
Stretching force was added continued for 1 hour at the cycle of 18% elongation for 1 second followed by 1-second relaxation.

Approximately 5.0 × 10^4^ ERM cells were seeded in flexible-bottomed culture plates coated with type I collagen (BioFlex Collagen; Flexcell International, McKeesport, PA, USA) and were cultured. After 1 week of culture, the ERM cells were stretched for 1 hour, with a cycle of 18% elongation for 1 second followed by 1-second relaxation, using an FX-4000 Flexercell Strain Unit (Flexcell International). The flexible cell-covered elastomer membranes were stretched by applying an oscillating vacuum to the underside of the membranes and the duration, amplitude, and frequency of the stretch applied were controlled by a computer (Figure 1). The vacuum manifold that held the plates was maintained at 37°C in a humidified incubator with 95% air and 5% CO_2_. The cells were collected at 0, 12, 24, 36, 48, and 72 hours after the initiation of the stretching force (stretching group). Cells without the stretching force were used as controls (control group).

Cell numbers were counted using a coulter counter (Beckman Coulter, Tokyo, Japan) at each of the time periods.

### 2.3. Immunofluorescence Observation

ERM cells were observed every day of the experiments using an ECLIPSE TS100 phase microscope (Nikon, Tokyo, Japan).

For standard fluorescence microscopic observations, cells in the dishes were washed with PBS, fixed with 4% paraformaldehyde for 20 minutes at room temperature and then were permeabilized in 0.1% Triton X-100 solution for 10 minutes. The cells were then washed twice with PBS. Nonspecific binding was blocked with 1% BSA. Filamentous actin (F-actin) stress fibers were visualized using FITC-conjugated phalloidin (1 : 100; Molecular Probes, Carlsbad) and were observed using a laser scanning microscope (LSM5 DUO, Carl Zeiss, Oberkochen). 

For investigating the effects of mechanical stretching in terms of protein levels, Immunofluorescence staining was carried out using a primary antibody against HSP70 (1 : 200 dilution; Abcam, Cambridge, UK) for 24 hours control and stretch group, VEGF (1 : 50 dilution; Abcam) for 48 hours control and stretch group, and OPN (1 : 40 dilution; Abcam) for 24 hours control and stretch group, a secondary antibody labeled with rhodamine (1 : 100 dilution; Invitrogen, Carlsbad, CA, USA) and were observed using a laser scanning microscope.

### 2.4. Actin Filament Direction Analysis

**Figure 2:**
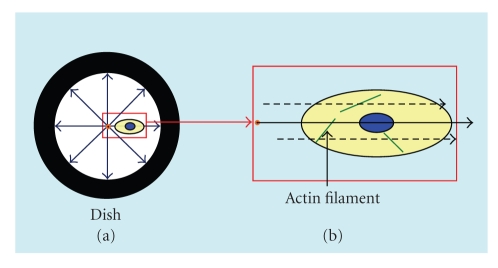
Stretching force is loaded equally by stretching machine (a). Stretching line was drawn from center point to the periphery of the culture dish through the center of cell nucleus in both groups. The angle made by this line and each of the actin filament in the cytoplasm on the Immunofluorescence picture was measured (b).

The line was drawn from center point to the periphery of the culture dish through the center of cell nucleus in both groups and the angle made by this line and each of the actin filament in the cytoplasm on the Immunofluorescence picture was measured as actin filament direction (Figure 2). For quantification, each angle created by actin filament against the line in the 5 cells on each of 5 culture dish in the both groups was analyzed using Image J analysis software (NIH, Bethesda, MD, USA).

### 2.5. mRNA Expression Analyses

In order to investigate the effects of mechanical stretching in terms of mRNA levels, quantitative real-time reverse transcriptase-polymerase chain reaction (RT-PCR) using a LightCycler was employed. Total RNA was extracted from each sample using the acid guanidinum thiocyanate/phenol-chloroform method. Cultured cells at each of the time periods were homogenized using a Bransonic sonicator (Branson, Danbury, CT, USA) and were solubilized in TRIzol Reagent (Invitrogen, Tokyo, Japan) and chloroform. Supernatants were obtained by centrifugation at 13200 rpm for 20 minutes at 4°C, added to isopropanol, stored for over 1 hour at −80°C, and were finally centrifuged at 13200 rpm for 20 minutes at 4°C. The precipitates were obtained by decantation and were washed with 70% ethanol. The RNA pellets were dissolved in RNAase-free water and kept at −20°C until use. Total RNA concentrations were measured by absorbance using a Nanodrop (ND-1000 Spectrophotometer, Scrum, Tokyo, JAPAN). Total RNA (1 *μ*g) was reverse transcribed and amplified in 20 *μ*L using an RT-kit (QIAGEN, MD, USA). cDNA synthesis was carried out using a Thermal Cycler (ThermoHybrid; ThermoBioAnalysis, Yokohama, Japan), and samples were incubated at 42°C for 15 minutes, heated to 99°C, and then quick-chilled to 5°C. Quantitative RT-PCR assays were performed using a LightCycler (Roche Molecular Biochemicals, Tokyo, Japan). Each reaction mixture considered 2 *μ*L LightCycler-FastStart DNA Master SYBR Green, 0.5 *μ*M of each primer, 5 mM MgCl_2_, and 2 *μ*L samples cDNA. Samples (total 20 *μ*L volume) were infused into glass capillaries. PCR was then carried out and mRNA expression of HSP70, VEGF, OPN, and the housekeeping gene GAPDH was measured using a LightCycler (Roche Diagnostics K.K.). The primer sequences for HSP70, VEGF, OPN, and GAPDH are shown in Table 1. The ratios of HSP70, VEGF, and OPN mRNAs were normalized against GAPDH. PCR data are reported compared to the corresponding control at 0 hour.

### 2.6. Statistical Analysis

Data from each experiment were statistically analyzed, and the assumption of homogeneity of variances and normal distribution of errors were tested for the response variables evaluated. Student's *t*-test was used to compare the actin filament direction and number of cells. One-way analysis of variance (ANOVA) and multiple comparison test (Bonfferroni's test) were used to compare mRNA levels for the control and stretching groups, and Student's *t*-test was used to compare various time periods (*P* = .05).

## 3. Results

### 3.1. Immunofluorescence Microscopic Observations of CK 19

**Figure 3:**
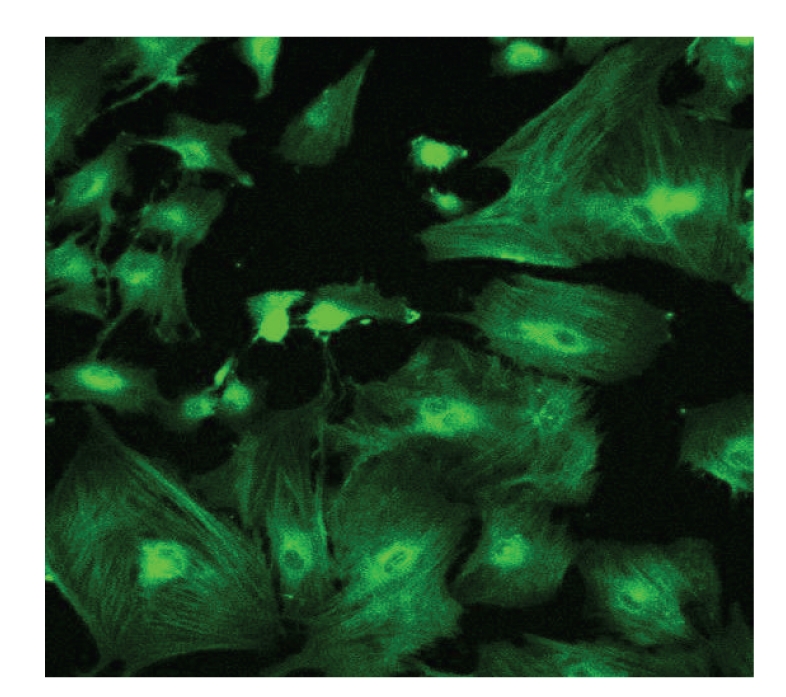
Immunofluorescence image of ERM cells using an antibody to cytokeratin 19 (CK 19). ERM cells were positive for CK 19.

MER cells were stained for cytokeratin 19 using an immunofluorescence technique (Figure 3).

### 3.2. Cell Proliferation Ratio

**Figure 4:**
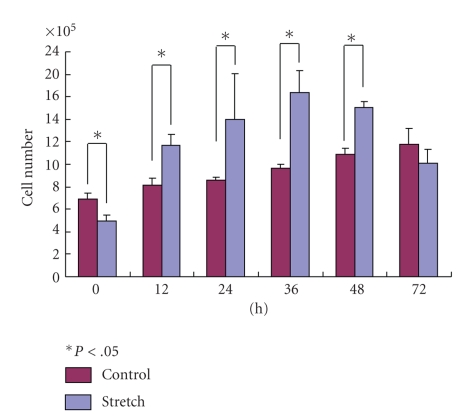
The numbers of cells in the stretching group were significantly less than the control group at 0 hour but were increased compared to the control group at 12, 24, 36, and 48 hours.

The numbers of cells in the stretching group were significantly less than in the control group at 0 hour. However, the numbers of cells in the stretching group were significantly increased compared to the control group at 12, 24, 36, and 48 hours (Figure 4).

### 3.3. Phase and Immunofluorescence Microscopic Observations and Actin Filament Direction

**Figure 5:**
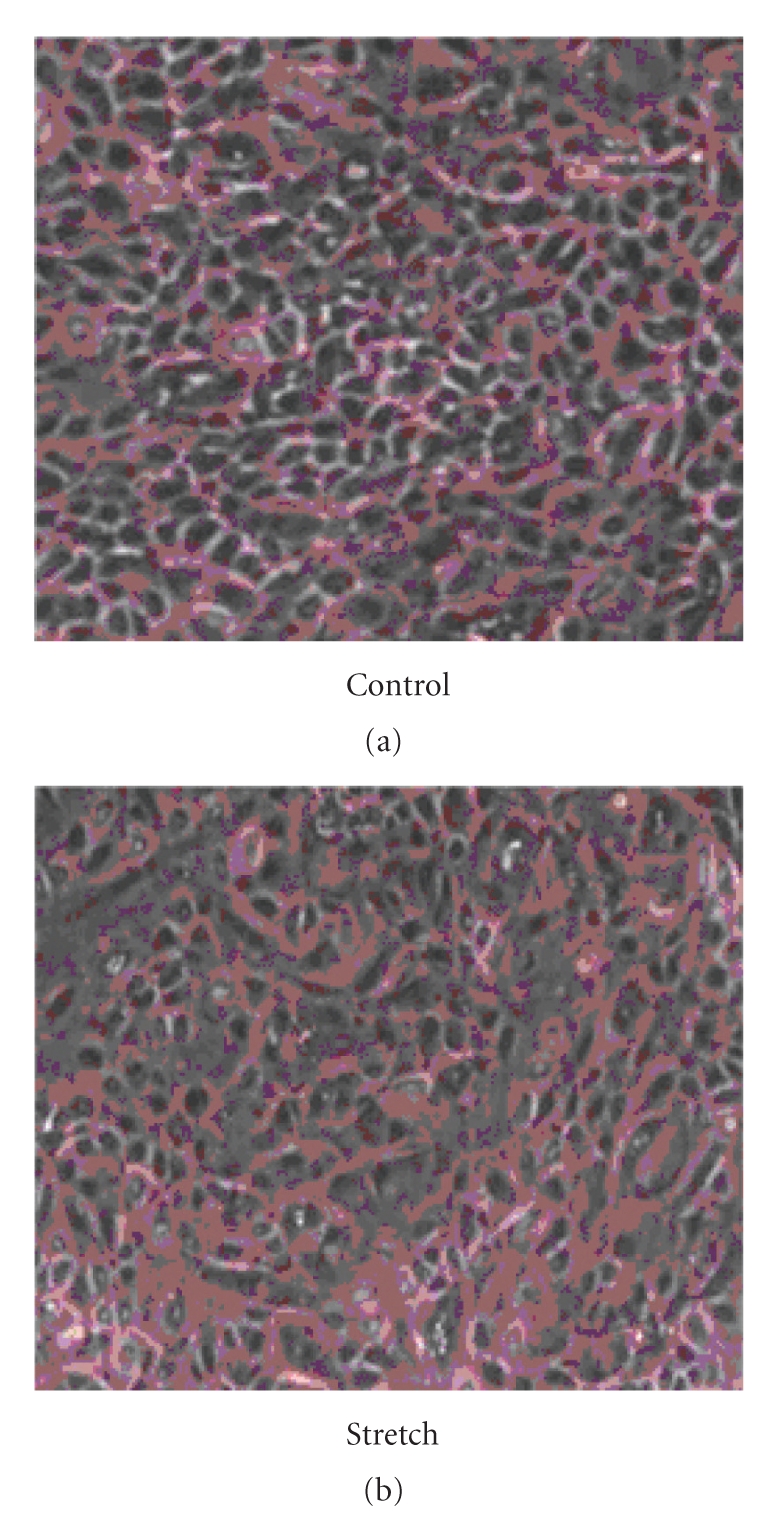
There were no remarkable morphological changes that could be seen between the control group and the stretching group at 0 hour.

There were no remarkable morphological changes that could be seen between the control group and the stretching group at 0 hour (Figure 5).

**Figure 6:**
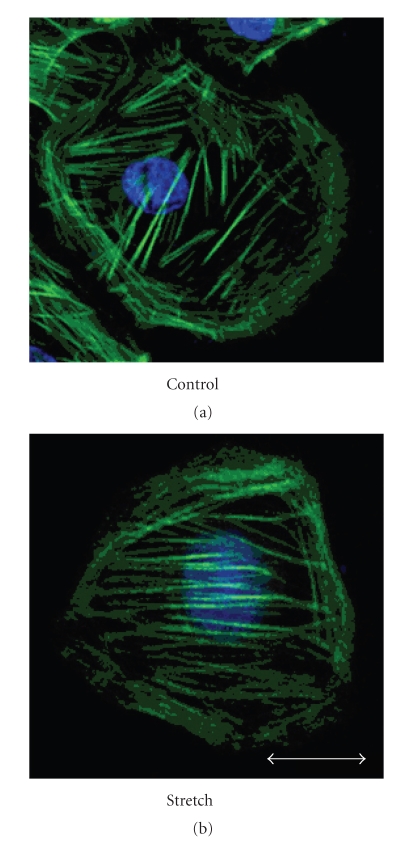
Actin filament directions of the cells in stretching groups were significantly lower degree than those of control groups (*P* < .05).

**Figure 7:**
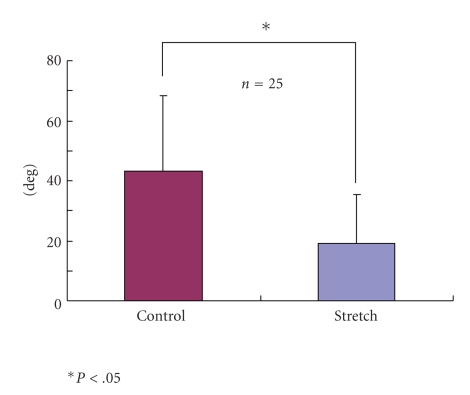
Actin filament directions of the cells in stretching groups were significantly lower degree than those of control groups (*P* < .05).

Actin filaments were randomly oriented in the cytoplasm of the control group (43.9 ± 5.2 degree), whereas in the stretching group, actin filaments were oriented parallel to the stretching direction (23.4 ± 1.9 degree) (Figures 6 and 7). There was a significant difference between the control groups and the test groups in terms of actin filament direction (*P* < .05).

### 3.4. Expression of HSP70-, VEGF-, and OPN-mRNAs

#### 3.4.1. HSP70-mRNA

**Figure 8:**
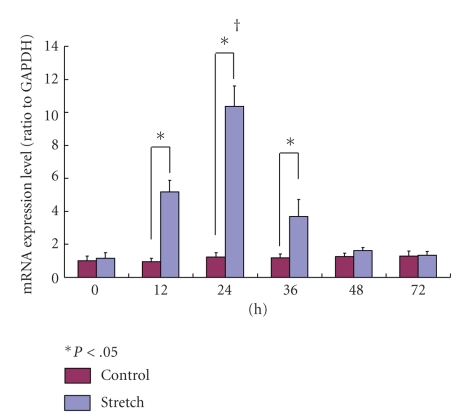
The expression of HSP70-mRNA in the stretching group was significantly higher than in the control group at 12, 24, and 36 hours (*P* < .05). The expression of HSP70-mRNA in the stretching group showed the highest value at 24 hours and decreased with time (†).

The expression of HSP70-mRNA in the stretching group was significantly higher than that in the control group at 12, 24, and 36 hours (*P* < .05). However there was no significant difference at 48 or 72 hours. The expression of HSP70-mRNA in the stretching group showed the highest value at 24 hours and decreased with time (Figure 8).

#### 3.4.2. VEGF-mRNA

**Figure 9:**
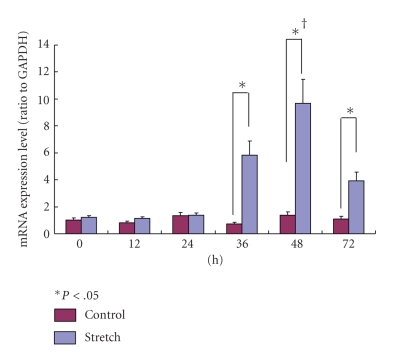
The expression of VEGF-mRNA in the stretching group was significantly higher than that is the control group at 24, 36, 48, and 72 hours (*P* < .05). The expression of VEGF-mRNA in the stretching group showed the highest value at 48 hours and decreased with time (†).

The expression of VEGF-mRNA in the stretching group was significantly higher than that in the control group at 36, 48, and 72 hours (*P* < .05). The expression of VEGF-mRNA in the stretching group showed the highest values at 48 hours and decreased with time (Figure 9).

#### 3.4.3. OPN-mRNA

**Figure 10:**
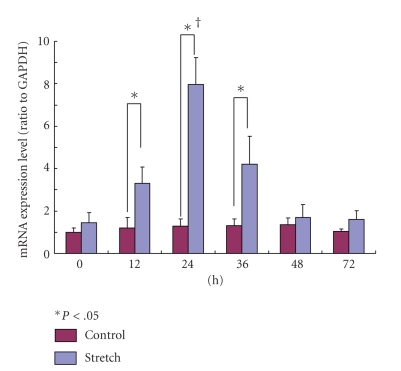
The expression of OPN-mRNA in the stretching group was significantly higher than the control group at 12 and 24 hours (*P* < .05). The expression of OPN-mRNA in the stretching group showed the highest value at 24 hours (†).

The expression of OPN-mRNA in the stretching group was significantly higher than that in the control group at 12, 24, and 36 hours (*P* < .05). The expression of OPN-mRNA in the stretching group showed the highest value at 24 hours (Figure 10).

The expression of HSP70-, VEGF-, and OPN-mRNAs was not significantly different in the control groups at any of the time periods.

#### 3.4.4. Immunofluorescence Assay for Protein Level of HSP70, VEGF, and OPN

**Figure 11:**
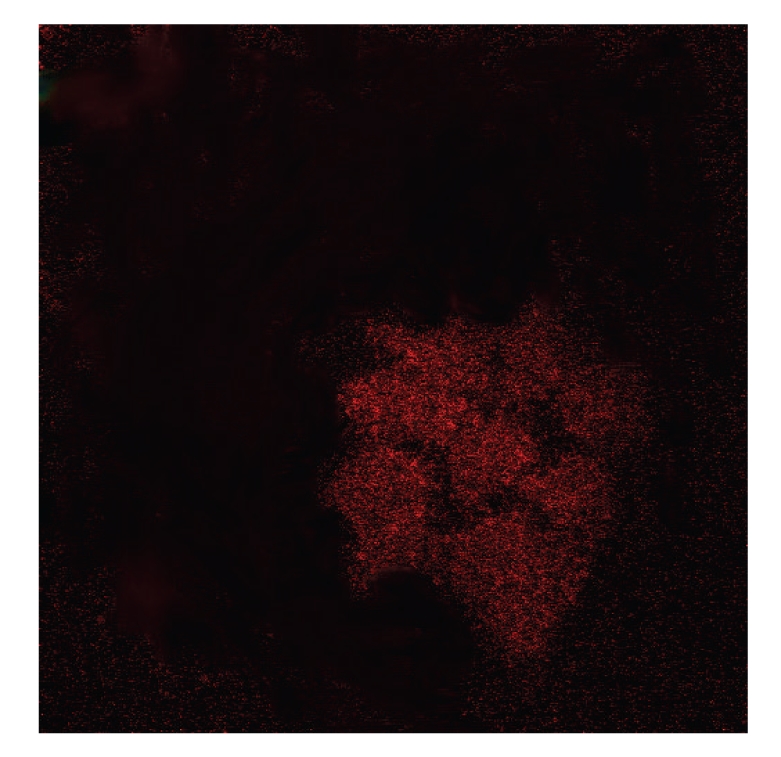
Immunofluorescence image of ERM cells using an antibody to HSP70 at 24 hours. ERM cells were positive for HSP70 at 24 hours stretch group.

**Figure 12:**
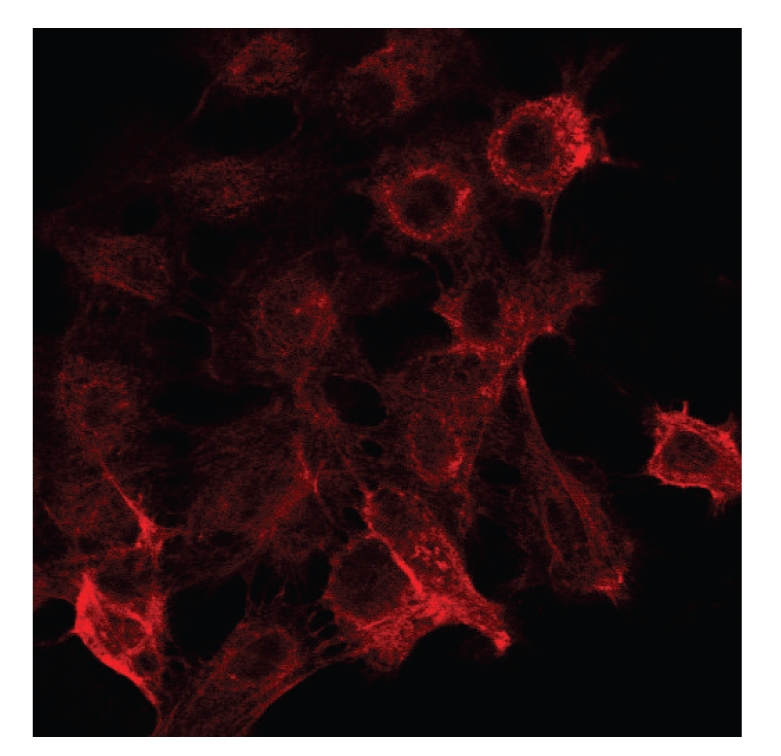
Immunofluorescence image of ERM cells using an antibody to VEGF at 48 hours. ERM cells were positive for VEGF at 48 hours stretch group.

**Figure 13:**
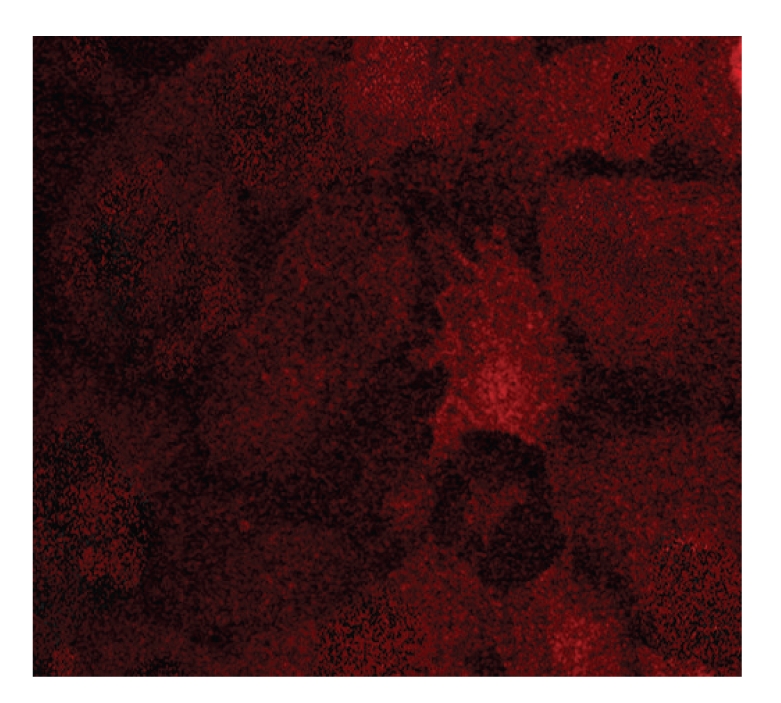
Immunofluorescence image of ERM cells using an antibody to OPN at 24 hours. ERM cells were positive for OPN at 24 hours stretch group.

MER cells were immunoreacted byr HSP70 at 24 hours stretch group (Figure 11), VEGF at 48 hours stretch group (Figure 12), and OPN at 24 hours stretch group (Figure 13).

## 4. Discussion

The Flexercell system used in this study assigned a specific stretching force to the cultured cells. Using this system, we applied intermittent mechanical stretching to produce 18% elongation at the periphery of each well and that elongated each cell body equally approximately 18%. This force is almost identical to those used in previous studies in which 18% cell stretching is a slightly higher level than the physiological force reported earlier [25].

In this study, we found that the direction of actin filaments in the experimental groups immediately after the stretching force was arranged depending on the attractive direction, while the filaments of the control groups were arranged randomly. However, Buck [29] and Dartsch [30] reported that the direction of actin filaments was arranged at a right angle to the stretching direction after 24 hours of stretching force and they concluded that the cells might be reacting against the stretching force. Most probably, the cells initially react to the stretching force after which they recover naturally.

Actin filaments are known to combine with HSP70 constantly [31], and it is thought that cytoskeleton proteins and stress proteins interact in stressed cells [32]. For this reason, it is thought that HSP70 may transmit the information in cooperation with integrins on the cell surface and cytoskeleton proteins when stretching forces are loaded.

In the stretching groups, more cell proliferation was observed than in the control groups. Brunette reported that mechanical stretching increases the number of epithelial cells synthesizing DNA [28]. Peake reported that deformed actin filaments promote cell proliferation [33]. Our study supports three reports and suggests that resistance of the ERM to the stretching stress may be high in the same way as fibroblasts [34].

Beere and coworkers reported that HSP expresses by a steady state combines with other proteins such as actin filaments [35, 36]. So, HSP guided by stress is thought to be important to the resistance of cells. In this study, the expression of HSP70 mRNA increased in the stretching group at 12, 24, and 36 hours, but not in the control group. This suggests that HSP70 probably reacts as autocrine to the damaged cells and maintains homeostasis of cells oneself.

It has been reported that VEGF resists the stresses of compression [37], hypoxia, and reoxygenation [19] and restores and maintains the increased activity of cells in the tissues. The expression of VEGF suggests that the cells have an important role in the homeostasis of the tissue in terms of nutrition. Ohshima et al. reported that ERM cells express VEGF, but periodontal ligament fibroblasts express VEGF in vitro [38]. In this study, the expression of VEGF was not detected in the control group; however, increased expression of VEGF at 24, 36, 48, and 72 hours was noted in the stretching group. As a result, the homeostasis of the periodontal ligament might be maintained by VEGF secreted by cells of the MER under conditions of stretching as paracrine.

OPN has been known as a marker for osteogenesis and differentiation of bone cells. ERM cells are also known to produce OPN in conjunction with cementogenesis and bone formation [39, 40]. Furthermore, it has been reported that periodontal ligament cells produce OPN under conditions of stretching. In addition, OPN is related to the increased activity of VEGF [41]. In this study, the expression of OPN was not remarkably changed in the control group; however, there was a significant increase in OPN mRNA at all time periods in the stretching group. The expression of OPN mRNA may control the hard tissue formation in the periodontal ligament. From these results, the expression of VEGF may occur first after which OPN expresses. This suggests that the ERM most probably maintains cementogenesis and osteogenesis against mechanical stress by expression of OPN.

## 5. Conclusion

ERM cells response against the stretching force by expressing HSP70, VEGF, and OPN.

## Figures and Tables

**Table 1:** Primer sequence.

		Sequence	Product Size
Hsp70	Forward:	5′-CGGACGAGTACAAGGTTGA-3′	206
	Reverse:	5′-CTCTTTCTCCGCCAACTG-3′	

VEGF	Forward:	5′-TTCCGAGAGTACCCCGATGA-3′	154
	Reverse:	5′-GGTGAGGTTTGATCCGCATA-3′	

OPN	Forward:	5′-ACCGATCCGACGAGTCTCA-3′	248
	Reverse:	5′-GGTACCATCCGTCTCCTCACTT-3′	

GAPDH	Forward:	5′-AGGGGCTCTCCAGAACATCA-3′	195
	Reverse:	5′-GCCTGCTTCACCACCTTCTT-3′	
